# Multifocal C-cell Hyperplasia and Marked Hypercalcitoninemia in a Diabetic Patient Treated With Glucagon-Like Peptide-1 Agonist With Concurrent Multinodular Goiter and Hyperparathyroidism

**DOI:** 10.7759/cureus.33384

**Published:** 2023-01-05

**Authors:** Sifan Zou, Alexandria D McDow, Zeb Saeed, Tieying Hou

**Affiliations:** 1 Department of Pathology and Laboratory Medicine, Indiana University School of Medicine, Indianapolis, USA; 2 Department of Endocrine Surgery, Indiana University School of Medicine, Indianapolis, USA; 3 Department of Endocrinology, Diabetes and Metabolism, Indiana University School of Medicine, Indianapolis, USA

**Keywords:** hyperparathyroidism, medullary thyroid carcinoma, calcitonin, glucagon-like peptide-1 receptor agonists, c cell hyperplasia

## Abstract

Thyroid C-cell hyperplasia (CCH) is divided into physiologic or reactive CCH and neoplastic CCH. Glucagon-like peptide-1 receptor agonists (GLP-1 Ra) is a group of medications used to treat type 2 diabetes that has documented C-cell stimulation effect in rodents, leading to subsequent CCH and medullary thyroid carcinoma (MTC) in rats and/or mice. Currently, there is no sufficient evidence supporting the association between GLP-1 Ra and human thyroid CCH and/or MTC. Here, we present a case of significant hypercalcitoninemia in a 53-year-old diabetic male patient receiving GLP-1 Ra treatment with concurrent multinodular goiter and hyperparathyroidism. Total thyroidectomy and central neck dissection revealed multifocal CCH involving bilateral thyroid lobes and several negative lymph nodes. Subsequent genetic testing did not detect germline mutation of *RET* gene. However, due to marked hypercalcitoninemia and massive thyromegaly, unsampled medullary thyroid microcarcinoma cannot be completely ruled out. The patient’s postsurgical calcitonin level was back to normal. Our case indicates the significant clinical value of monitoring serum calcitonin levels in patients receiving GLP-1 Ra, especially in presence of other thyroid and/or parathyroid pathology that may be associated with increased calcitonin and/or CCH. Literature regarding the association between GLP-1 Ra and CCH is also reviewed.

## Introduction

C cells are neural crest-derived cells that are concentrated in the lateral aspects of the upper two-thirds of bilateral thyroid lobes. They secrete calcitonin, a 32 amino acid peptide hormone that regulates calcium homeostasis. Thyroid C-cell hyperplasia (CCH) is characterized by the proliferation of C cells in between follicular space that is identified on an H&E-stained slide and/or by immunostaining of calcitonin. It is divided into physiologic (or reactive) CCH and neoplastic CCH. Physiologic CCH has been observed in patients with Hashimoto’s thyroiditis [1], multinodular goiter [2], follicular thyroid neoplasm [3], and hyperparathyroidism [4]. Neoplastic CCH is considered a precursor of familial medullary thyroid carcinoma (MTC) that is associated with germline RET gene mutation [5].

Glucagon-like peptide-1 receptor agonists (GLP-1 Ra) are a group of medications that are used to treat type 2 diabetes (T2DM) and some of GLP-1 Ra are also approved for the treatment of obesity. In rodents, injection of GLP-1 Ra has been shown to cause CCH and elevated serum calcitonin levels [6]. Although most literature has shown that long-term use of GLP-1 Ra in humans has no effect on serum calcitonin level and do not cause C-cell-derived neoplasm [7], some recent data analysis reveals increased risk for thyroid cancer in patients receiving GLP-1 Ra treatment for T2DM [8]. Here, we report a case of multifocal CCH and prominent hypercalcitoninemia in a diabetic patient treated with GLP-1 Ra with concurrent multinodular goiter and hyperparathyroidism. We also reviewed the literature regarding the effect of GLP-1 Ra on thyroid C-cell pathology.

## Case presentation

The patient is a 53-year-old male with a history of uncontrolled T2DM, hypertension, chronic kidney disease, and morbid obesity. In addition, he had nontoxic multinodular goiter and secondary hyperparathyroidism due to chronic kidney failure. Ultrasound showed bilateral massive thyromegaly with a left substernal extension which caused tracheal deviation to the right and mild tracheal narrowing. Fine needle aspiration of three thyroid nodules resulted in benign colloid nodules. He had been treated with Glucagon-like peptide-1 receptor agonist (GLP-1 Ra) for six months before pursuing surgical intervention of multinodular goiter. Presurgical workup revealed normal thyroid function and increased PTH level to 222 pg/mL with a normal calcium level of 8.8 mg/dL in the setting of stage IV chronic kidney disease. Interestingly, his calcitonin level was also significantly elevated to 140 pg/mL (reference range: 0-7.5 pg/mL). His preoperative neck CT reveals slightly enlarged lymph nodes favoring reactive change. Also, lymph node mapping by ultrasound showed benign appearing cervical lymph nodes with prominent fatty hila, likely representing reactive adenopathy.

**Figure 1:**
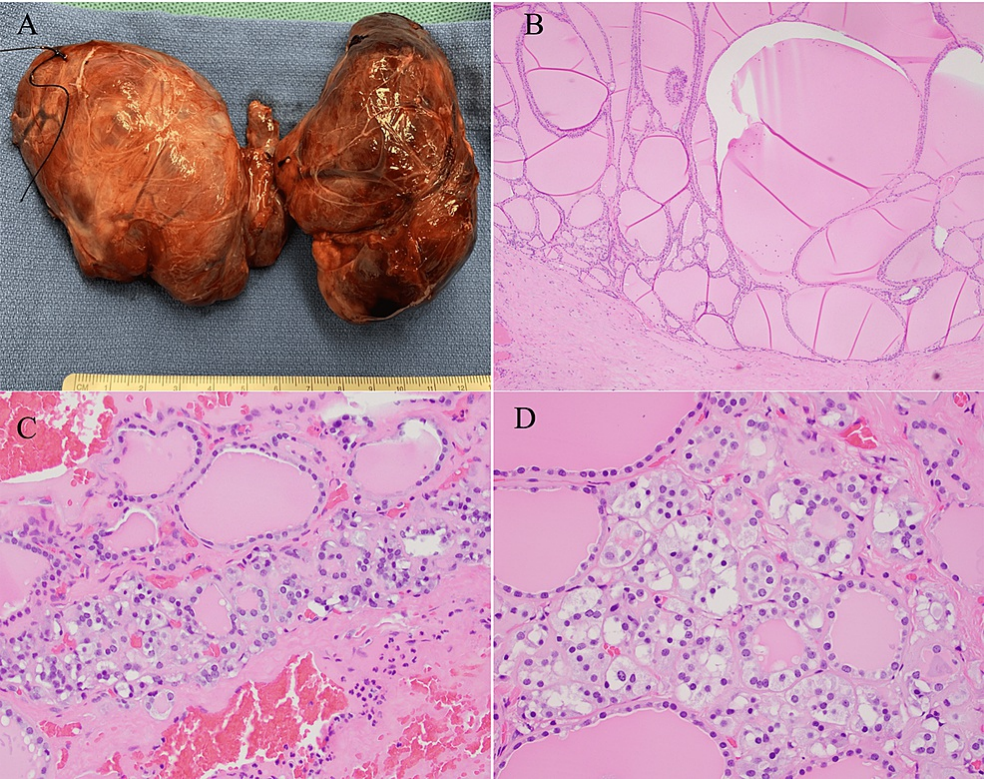
Gross and microscopic findings from total thyroidectomy specimen (A) Gross examination of massive thyromegaly. (B) Thyroid with nodular hyperplasia (200x). (C) C-cell proliferation (CCH) in the intrafollicular space (200x). (D) High power field of CCH demonstrate small, round nuclei, and fine chromatin with clear or granular cytoplasm. Nuclear size is similar to or slightly larger than follicular cells. No mitotic figure, cytologic atypia or desmoplastic change is identified (400x).

**Figure 2:**
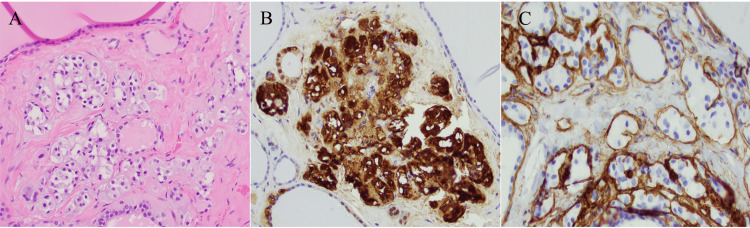
H&E examination and immunohistochemical staining CCH identified on H&E slide (A) was stained strongly and diffusely positive for calcitonin (B). Collagen type IV immunostain showed intact basement membrane layer (C). (200x for A-C).

Given multinodular goiter with calcitonin elevation, total thyroidectomy with central neck dissection was performed. Left inferior parathyroid was found to be enlarged during surgery and was also excised. Grossly, the left and right thyroid lobes measured 10 cm in the greatest dimension for each lobe. Isthmus was 6 cm in size (Figure 1A). Sectioning revealed a multinodular surface with cystic changes. No discrete lesion or nodule was grossly identified. Microscopically, multifocal CCH was identified in 6 sections out of 33 sections submitted from the left and right lobes ranging from < 1.0 mm to 1.5 mm in the greatest dimension in a background of nodular hyperplasia. The nuclear size of C cells was similar or slightly larger to the surrounding follicular cells with granular or pale cytoplasm. No significant cytologic atypia, mitotic figure, or desmoplastic change was identified (Figures 1A-1D, 2A-2C). Immunostain for calcitonin was strongly and diffusely positive in CCH. Immunostain for collagen type IV highlighted the intact basement membrane layer (Figures 2A-2C). Total four benign lymph nodes were identified in central compartment dissection. The left inferior parathyroid revealed slightly hypercellular parathyroid tissue.

The patient recovered well from surgery and his postoperative calcitonin dropped to less than 0.2 pg/mL about four weeks after the surgery (Table 1). PTH level normalized to 26 pg/mL. The patient was referred to genetic testing due to pathologic findings. No germline mutation of RET gene was detected. Due to the concern about the relationship between GLP-1Ra and CCH, the patient’s treatment for diabetes was switched to insulin. However, the patient’s glucose level was poorly controlled with insulin and the patient became frustrated with the inability to lose weight despite adhering to a very low carbohydrate/ketogenic diet. After a long discussion with the patient, he was started with Mounjaro (tirzepatide) injection, a new glucose-dependent insulinotropic polypeptide (GIP) and GLP-1Ra recently approved by FDA for the treatment of adults with T2DM [9]. The patient has been closely monitored for serum calcitonin level every three months and for neck ultrasound every six months. Both his calcitonin levels and neck ultrasound remained normal in the most recent follow-up in eight months after surgery.

**Table 1:** Patient’s related laboratory results

	Before GLP-1 Ra	Presurgical	Postsurgical	Reference ranges
TSH	0.417	1.111	1.958	0.4-4.2 mcU/mL
Total T3	Not test	86	Not test	82-179 ng/dL
Free T4	Not test	0.8	1.0	0.6-1.5 ng/dL
Calcium	9.1	9.1	9.1	8.5-10.5 mg/dL
Creatinine	1.42	2.85	2.87	0.80-1.40 mg/dL
PTH	117	222	26	10-65 pg/mL
Calcitonin	Not test	140	< 2.0	0-7.5 pg/mL

## Discussion

The definition of CCH is not well established, and several diagnostic criteria have been proposed including > 50 C cells in one low power field (100x), > 40 cells/cm^2^, > 50 cells in 50 low power field (100x), or > 50 calcitonin-positive cells in three low power field (100x). The presence of different explanations is caused by a lack of complete understanding of the distribution and localization of physiologic C cells that tend to vary with age, sex, calcium level, and thyroid pathology. Neoplastic CCH is well established as a precursor of familiar MTC that is most often seen in multiple endocrine neoplasias 2A/2B (MEN 2A/2B). Molecular analysis of microdissected CCH from thyroidectomy specimens of patients with MEN 2A demonstrated monoclonality, down-regulation of apoptosis genes, and multiple tumor suppressor gene abnormalities, which support they are intraepithelial neoplasia [5]. Perry, et al. [10] reported that physiologic/reactive CCH was biologically and morphologically different from neoplastic CCH. Their study showed that the former was not readily identified on H&E slides, required calcitonin immunostain, and lack of cytologic atypia seen in neoplastic CCH. Other authors, however, believe that pathologists cannot reliably distinguish physiologic CCH from neoplastic CCH by morphology [11]. In Verga et al.’s study [12], when stimulated calcitonin level was > 50 pg/mL in patients with multinodular goiter, the morphology and distribution pattern of C cells were similar to those observed in familial MTC cases. None of these patients were found to harbor germline or somatic mutation of RET gene. In their opinion, only molecular analysis of RET gene mutation can distinguish physiological CCH from neoplastic CCH.

The architecture of CCH is divided into focal, diffuse, and nodular patterns. The upper limit of CCH and the lower limit of thyroid medullary microcarcinoma are ill-defined. The assessment of any defect in the basement membrane by immunohistochemical staining for collagen type IV may help distinguish these two situations [13]. In our case, lack of cytologic atypia and desmoplastic change, as well as intact basement membrane demonstrated by collagen IV staining support the diagnosis of CCH instead of microcarcinoma. However, due to the massive thyroidectomy and the significantly elevated calcitonin level over 100 pg/mL, potentially unsampled microcarcinoma cannot be completely ruled out. Medullary thyroid microcarcinoma (defined as < 10 mm) is treated by total thyroidectomy and central compartment dissection, which has been done for our patient. The positive nodal status ranges widely from 5% to 43% in sporadic medullary thyroid microcarcinoma and the risk of nodal metastasis is increased with the increase in tumor size [14]. In Elisei et al.’s study, distant metastasis was only seen in 1.3% of patients with medullary thyroid microcarcinoma including 126 hereditary and 107 sporadic types [14]. None of the patients died of the disease during their follow-up. 

Although not well understood, two pathogenetic mechanisms have been proposed for physiologic CCH. Chronic thyroid-stimulating hormone (TSH) overstimulation and the interaction between follicular cells and C cells may contribute to reactive CCH observed in patients with thyroid pathology including lymphocytic thyroiditis, multinodular goiter, and follicular neoplasm [1-3]. The other mechanism is CCH in an attempt to secret more calcitonin to control the hypercalcemia developed in cases with hyperparathyroidism [4]. In addition to these two factors, our patient had also been treated with GLP-1 Ra for his T2DM. GLP-1 Ra is a class of mediation that works by mimicking the function of natural GLP-1. Long-term exposure to GLP-1 Ra in rodents was found to stimulate calcitonin secretion, upregulate calcitonin mRNA, and lead to subsequent CCH and C-cell neoplasm [15]. This effect was not observed in humans or monkeys probably due to a lack of or very low mRNA level of GLP-1 R in human or nonhuman primates compared to mice and/or rats [15]. Although Gier et al. [16] reported the concurrent immunoexpression of GLP-1 R and calcitonin in human CCH, MTC, and a subset of papillary thyroid carcinoma as well as normal human thyroid tissue, this result was later on challenged by other authors because polyclonal rabbit antibody used in the study may have a cross reaction with another G-protein receptor sharing homology with GLP-1 R. Several randomized prospective clinical trials showed that GLP-1 Ra, liraglutide and exenatide, did not increase serum calcitonin levels in two to three years follow up [7,17]. In addition, Cao et al. performed a meta-analysis of 37 clinical trials that revealed no increased risk of thyroid or pancreatic cancer in the GLP-1 Ra-treated group compared to the control [18]. However, a few other studies have reported a possible association between GLP-1 Ra and thyroid cancer. In 2011, soon after the authorization of GLP-1 Ra, an examination of the US Food and Drug Administration Adverse Event Reporting System (FDA FAERS) from 2004 to 2009 revealed that the use of exenatide or sitagliptin increased the odds ratio for thyroid cancer to 4.73 or 1.48, respectively [19]. Analysis of the European pharmacovigilance database also demonstrated increased proportional reporting ratios (PPRs) for thyroid cancer, medullary thyroid carcinoma, and thyroid neoplasm in patients treated with GLP-1 Ra [8]. Recently, Wang et al. reported that GLP-1 Ra was associated with a higher risk for thyroid cancer (adjusted odds ratio: 4.33) after analysis of the FDA FAERS database from 2005 to 2019, although the subtypes of thyroid cancer were not specified in this study [20].

## Conclusions

In conclusion, we reported a case of multifocal CCH and marked hypercalcitoninemia in a diabetic patient treated with GLP-1 Ra with concurrent multinodular goiter and hyperparathyroidism. The direct relationship between GLP-1 Ra and elevated calcitonin level/CCH is difficult to prove because of coexisting thyroid pathology and abnormal parathyroid function, which have also been reported to be associated with CCH. However, our report suggests that there is a clinical significance in monitoring serum calcitonin levels in patients receiving GLP-1 Ra treatment especially when other conditions that may increase the risk for CCH or C-cell neoplasm coexist. The long-term effects of GLP-1 Ra on human thyroid pathology need to be further investigated.
